# Biological Complexities in Radiation Carcinogenesis and Cancer Radiotherapy: Impact of New Biological Paradigms

**DOI:** 10.3390/genes3010090

**Published:** 2012-01-20

**Authors:** Hossein Mozdarani

**Affiliations:** Department of Medical Genetics, Faculty of Medical Sciences, Tarbiat Modares University, Tehran P.O.Box 14115-111, Iran; E-Mail: mozdarah@modares.ac.ir

**Keywords:** radiation carcinogenesis, genome instability, radiosensitivity, radio-adaptation, bystander effect

## Abstract

Although radiation carcinogenesis has been shown both experimentally and epidemiologically, the use of ionizing radiation is also one of the major modalities in cancer treatment. Various known cellular and molecular events are involved in carcinogenesis. Apart from the known phenomena, there could be implications for carcinogenesis and cancer prevention due to other biological processes such as the bystander effect, the abscopal effect, intrinsic radiosensitivity and radioadaptation. Bystander effects have consequences for mutation initiated cancer paradigms of radiation carcinogenesis, which provide the mechanistic justification for low-dose risk estimates. The abscopal effect is potentially important for tumor control and is mediated through cytokines and/or the immune system (mainly cell-mediated immunity). It results from loss of growth and stimulatory and/or immunosuppressive factors from the tumor. Intrinsic radiosensitivity is a feature of some cancer prone chromosomal breakage syndromes such as *ataxia telangectiasia*. Radiosensitivity is manifested as higher chromosomal aberrations and DNA repair impairment is now known as a good biomarker for breast cancer screening and prediction of prognosis. However, it is not yet known whether this effect is good or bad for those receiving radiation or radiomimetic agents for treatment. Radiation hormesis is another major concern for carcinogenesis. This process which protects cells from higher doses of radiation or radio mimic chemicals, may lead to the escape of cells from mitotic death or apoptosis and put cells with a lower amount of damage into the process of cancer induction. Therefore, any of these biological phenomena could have impact on another process giving rise to genome instability of cells which are not in the field of radiation but still receiving a lower amount of radiation. For prevention of radiation induced carcinogenesis or risk assessment as well as for successful radiation therapy, all these phenomena should be taken into account.

## 1. Introduction

There is now a huge bulk of evidence that shows ionizing radiations are potent carcinogens and have potential to elevate the frequency of naturally occurring cancers. It was only a few years after the discovery of X-rays by Roentgen and radioactivity by Becquerel that a strong correlation was observed between exposure to ionizing radiation (IR) and the development of cancer. The first radiation induced cancer was reported to arise in an ulcerated area of the skin of a dentist who had held the film in the mouth of patients. The first report of leukemia, occurring in five radiation workers, appeared in 1911 [1]. As the use of ionizing radiation grew, more and more malignancies have appeared. We cannot forget skin cancers which occurred in many pioneers in radiation science. Marie Curie and her daughter Irene both are thought to have died from leukemia. Radio dermatitis leading to malignancies was common place in the hands of radiation workers. We also cannot forget the radium girls who suffered from bone cancer, a very rare malignancy in humans. The massive rise in the incidence of thyroid cancer—a very rare cancer in children—among children in the Ukraine and in Belarus following the Chernobyl accident means that “each such cancer” is radiation induced with a probability of >90% [2]. The mean dose for radiation-induced thyroid cancers in Belarusian children was <0.2 Gy [2]. Observations of radiation related cancers in different tissues show radiation as a universal carcinogen. The cancers that are induced by radiation are of the same histological types as the ones that occur spontaneously.

The advances in nuclear physics have led to new applications of nuclear energy in modern life. The growing use of ionizing radiation in medicine both for imaging and therapy has led to the exposure of many individuals to low-level ionizing radiation, occupationally or at general population level. The growing number of nuclear power plants together with the large number of employees working in these places and nearby residents, are also a major concern for exposure of a large section of the population to ionizing radiation. Apart from the occupational exposure, many people live in high natural background radiation areas in many countries. An extensive study of the effects of low-level irradiation over a relatively long period of time was carried out by the US National Academy of Sciences [3]. Some research groups suggest that the effects of low-level radiation exposure may be even more severe than those at higher values.

The biological effects of ionizing radiation in mammalian cells include gene mutation, chromosomal rearrangement, cellular transformation, cell death and carcinogenesis. Cancer induction is the most important somatic effect of low dose radiation. It is commonly stated that “any radiation dose, no matter how small, can cause cancer.” Therefore radiation carcinogenesis follows a linear-no threshold hypothesis. Thus the cancer risk is not zero regardless of how small the dose is. A fundamental challenge in radiation research related to human health is to predict the biological impact of exposure to low dose (<0.1 Gy) ionizing radiation. Excess cancers have been observed in the Japanese atomic-bomb survivors at doses of 0.1 to 4 Gy. Below 10 cGy risks of health effects are either too small to be observed or are nonexistent.

For many years, the nucleus, specifically the DNA, has been the principal target for the biological effects of radiation. Following irradiation, the initial radiation induced DNA damage is converted into a mutation or chromosomal aberration during subsequent impaired DNA repair and is expressed by the irradiated cell and its progeny. In the last two decades, the understanding of the cellular and molecular processes that eventually lead to the different types of cancers, has increased explosively. Biological modifying factors have emerged and have already entered into some models of radiation carcinogenesis, although the large numbers of complex and modifying factors have not yet been assessed quantitatively and the predictive value of the models is very limited. There is a large body of evidence showing that radiation has a stimulating effect on a number of biological processes and can induce resistance against higher doses of ionizing radiation. Most of these studies were performed in *in vitro* conditions and now, there is little doubt that the *in vitro* pretreatment of human lymphocytes with tritiated thymidine or with low doses of X-rays, makes these cells less susceptible to cytogenetic damage by subsequent high acute doses of X- or gamma-rays [4,5,6]. The induction of a radioprotective mechanism by low doses has important implications for human health. On the other hand, about 10% of the general population show a degree of radiosensitivity in terms of cytogenetic damage. It is now recognized that development of cancer is a much more complex process than was originally thought. The role of “bystander effects” signaling between neighboring cells about their radiation experiences [7], is now recognized to be an important, albeit poorly understood factor. These known phenomena make the linear non threshold (LNT) hypothesis less valid for radiation carcinogenesis, especially for doses lower than 10 cGy.

Further evidence of active cellular processes initiated by radiation *i.e*., genomic instability, bystander effects and adaptive responses, should now be considered as new paradigms that contribute to active cellular processes. In spite of the fact that the underlying mechanisms of genomic instability, bystander effects and adaptive responses each remains to be understood, some investigators are trying to unify these low-dose phenomena and show their impact on low dose radiation carcinogenesis. Here in this review these known and proposed interrelationships will be discussed.

## 2. Radiation Carcinogenesis

Carcinogenesis, a stochastic effect of ionizing radiation, is the main delayed somatic effect of ionizing radiation. The malignancies induced by irradiations are indistinguishable from those occurring from many other causes. It is a well-established fact that low-dose effects of IR are more difficult to measure than high dose effects. Epidemiological studies usually lack sufficient statistical power to determine health risks arising from very low-dose exposures. In this situation, studies of fundamental mechanisms involved can help to understand and assess short and long term effects of low-dose IR and to evaluate the associated risks for human health, and in particular the risk of cancer.

Research on radiation carcinogenesis during the past two decades has focused on the cellular and molecular mechanisms of radiation effects in mammalian cells. Ionizing radiation has DNA as a critical target, directly or indirectly via formation of reactive oxygen species (ROS) which give rise to various types of DNA lesions, including single-strand breaks and double-strand breaks (DSBs), and various types of base damage as well as DNA-DNA and DNA-protein cross links. The formation of ROS produces not only DNA strand breakages, but also might act as a signaling event leading to the release of cytokines or epigenetic changes, or trigger DNA repair machinery. Many more parameters are involved in responses at low doses than at high doses, and different pathways are activated. At low doses, however, taking into account all these cellular responses, non-linear responses are obtained that are not compatible with the LNT concept. Indeed, several lines of evidence from molecular, biochemical and biological studies appear to suggest future changes in radiobiological paradigms and concepts which concern low-dose IR effects and the evaluation of human health risks.

All primary lesions induced in the DNA are subjected to cellular repair processes; however, the unrepaired or misrepaired lesions may give rise to chromosomal aberrations (CA). Although double-strand breaks will be induced even by very low doses of radiation, they may be repaired very effectively by either one of the two different repair mechanisms namely, homologous recombinational repair (HRR) and non-homologous end joining (NHEJ) [8,9]. HRR is able to restore the original sequence of DNA DSB leading to a lower risk of generation of deletions and insertions at the site of DSB. HRR is limited to those DSBs which are located within sequences having at least one further and undamaged copy somewhere else in the genome [10]. HRR activity is non mutagenic at sites of DSBs but only few breaks are involved with this type of repair [11]. Various types of DNA repair proteins are involved in HRR (namely, RAD52, Rad51B, Rad51C, Rad51D, Xrcc2, Xrcc3 and BRCA2) [12]. NHEJ is subject to a high risk of generation of *de novo* mutations at the sites of DSBs. Thus susceptibility to mutagenesis is a direct consequence of the NHEJ system joining DNA free ends. The main proteins involved in NHEJ pathways are DNA PKcs, ATM and MRN complex [11]. Several groups have demonstrated that proteins associated with the repair of DNA may contribute to the radiation induced instability process, such as DNA PKcs [13] and p53 [14,15]. Furthermore, protection of telomeres by certain DNA repair proteins in the non-homologous pathway of double strand break (DSB) rejoining has been shown [13,16]. Cells having mutations in genes coding for these proteins are subjected to multiple forms of instability-defective DSB repair, chromosomal end-to-end fusions, and joining of unprotected telomeres to radiation-induced DSB ends [17,18,19]. Therefore, these attenuated normal gene functions may contribute to the initiation of instability, as seen in some heritable genetic disorders such as *ataxia telangiectasia* (AT), Nijmegen breakage syndrome and others.

Accumulating evidence points to the unrepaired DSBs as the major lesion in the cellular, chromosomal, mutagenic and oncogenic effects of ionizing radiation. Radiation-induced instability endpoints have been shown to be manifested as chromosomal alterations, micronuclei, cell transformation, gene amplification, apoptosis, and sister chromatid exchange, *etc*. The induction of the unstable CA following the exposure of cells at the G1 phase and chromatid-type aberrations after the irradiation of cells at the G2 phase of the cell cycle, is quite characteristic of ionizing radiation [20,21,22,23]. The studies on derived mutants of mammalian cells which are deficient in the repair of DSBs show a high chromosomal sensitivity [24,25,26] and also, the generation of DSB in the DNA of the cells by the restriction endonucleases [27,28] strongly argues in favor of the DSB as the lesion, leading to CA in the irradiated cells. The chromosomal alterations represent an important portion of the total spectrum of damages induced in the genetic materials of cells by radiation. The information obtained from numerous cytogenetic studies has provided the relevance of such damage in determining the risk of the delayed effects on the exposed population, as well as the mechanisms of the induction, accumulation, persistence and elimination of CA.

Bender and his colleagues (1974) [20] proposed that the single DNA molecule has to be broken (DSB) before interaction can take place with another DSB. This event leads to a misjoined structure type such as a dicentric chromosome or a ring with an associated acentric fragment or a stable type such as reciprocal translocation. These induced aberrations allow cells to continue proliferation with over expression of truncated oncogene leading to neoplastic transformation. As an example, a translocation between chromosomes 9 and 22 t(9;22)(q34;q11) in chronic myeloid leukemia resulted in Philadelphia chromosome. This translocation of the ABL proto-oncogene from its normal site on chromosome 9q34 to chromosome 22q11 forms a fusion hybrid gene with the breakage cluster region. Expression of the hybrid gene leads to the loss of cell cycle control, promotion of cell growth and inhibition of DNA repair leading to genomic instability. A similar case is Burkitt’s lymphoma characterized by a chromosome 8;14 translocation [29]. Translocations which are frequently seen in tumor cells are considered to be the result of recombinational events, thus the control of DSBs by cells ensures genomic stability.

In the context of solid tumors, rearrangements of the *RET* (rearranged during transfection) gene, known as *RET/PTC* rearrangements occur in papillary thyroid carcinoma (PTC) [30]. More than 10 such rearrangements have been described; *RET/PTC1*, *RET/PTC2* and *RET/PTC3* account for most of the rearrangements found in PTC [31,32]. Reports after the Chernobyl power plant explosion indicate a sharp increase in the incidence of pediatric thyroid papillary cancer with a high prevalence of solid-follicular tumors. In all these studies analysis of these PTCs show a strong correlation between the solid variant PTC and the *RET/PTC3* activation [33,34]. However, in a study of thyroid nodules in individuals with no history of radiation exposure, Belarusian subjects exposed to post Chernobyl radioactive fallout and individuals exposed to external irradiation of head and neck, Elisei *et al*. (2001) have indicated that the presence of *RET/PTC* rearrangements in thyroid cancer was not higher in radiation induced tumors compared with those naturally occurring [35].

It has been known for a long time that there is an association between chromosomal rearrangements and tumor formation. Several reports have shown that CA frequencies are increased prior to the clinical manifestation of cancer [36,37] and the translocations appear to be involved in some human malignancies [38]. Numerous reports indicate that most neoplasms are associated with chromosomal rearrangements [29], which can lead to the activation of proto-oncogenes and the elimination of the tumor suppressor genes, therefore representing an important mechanism of tumorigenesis. It is well documented that the CA assays is one of the most sensitive and specific assays for use in the identification of carcinogens and non-carcinogens [39,40,41].

Oncogenic transformation has been demonstrated in many studies to date to be an integral stage in carcinogenesis. Oncogenes are produced by mutational changes in proto-oncogenes present in the normal genomic DNA of cells. Despite the fact that radiation is known to cause double-strand breaks and genomic rearrangements, the mechanisms leading to proto-oncogene activation appear to be much more complex than just random DNA double-strand breaks, formation and reunion. Many of the genes, which if mutated, cause progression of the carcinogenic process by a further step have been characterized with regard to their normal function and the functional impairment that occurs as a result of the mutation. Gene amplification might also play an important role in oncogenic transformation. Studies on the organization of the amplified DNA in tumor cells have suggested that a single DNA DSB can trigger a cascade of events leading to amplification of a gene in the genome. Therefore a gene involved in DNA damage response and in DNA damage repair can play a role in controlling the amplification process. It has been shown that defects in the DSB repair mechanism can increase the frequency of gene amplification [42].

Loss of function of tumor suppressor genes is assumed to be the first step in the majority of solid cancers, whereas in the development of leukemia and lymphoma the first step appears to be the activation of a proto-oncogene into an oncogene, e.g., by translocation of a promoter besides the active site of a normally repressed growth promoting gene site. About 50 gene specific chromosome translocations have been characterized at the molecular level in human leukemias and lymphomas [43]. Since loss of heterozygosity mutations are characteristic consequences of DNA double-strand breaks, radiation carcinogenesis researches have concentrated on this type of mutation and thus on the role and fate of DNA double-strand breaks.

Although the relevance of radiation-induced persistent genomic instability to neoplastic development remains to be established [43], direct induction of loss of heterozygosity as a consequence of radiation-induced complex double-strand breaks may play only a minor role in radiation carcinogenesis compared with the small mutations which also occur spontaneously and which may become more frequent as a result of radiation-induced genomic instability. Therefore genome instability might be the underlying mechanism of various cellular responses to ionizing radiation.

## 3. Impact of Radiosensitivity on Carcinogenesis and Cancer Therapy

Several DNA damage processing and repair pathways constitute a guard system that protects cells against genetic instability and tumorigenesis [44,45,46]. Both impaired DNA repair and genome instability are considered as factors underlying increased susceptibility to malignancy [47,48]. It has been suggested that individuals who are genetically susceptible to cancer manifest the impaired DNA damage identification and repair by exhibiting increased DNA radiosensitivity [49].

The biological importance of genomic instability and DNA repair mechanisms in cancer development are particularly well illustrated by several heritable genetic disorders known as chromosome breakage or chromosomal instability syndromes. These chromosome breakage syndromes are characterized by various defects in DNA repair, predisposition to various forms of malignancies and increased radiosensitivity [23,45,50,51,52]. The chromosome breakage syndromes such as *ataxia-telangiectasia*, Nijmegen breakage syndrome, *ataxia-telangiectasia*-like disorder, Bloom syndrome, Werner syndrome and Fanconi anemia are human autosomal recessive diseases characterized by inherited chromosomal instability and cancer predisposition [48,53,54]. Higurashi and Conen (1973) [55] were one of the first to report high levels of gamma-ray induced chromosomal damages in lymphocytes of patients with AT, Fanconi’s anemia (FA) and Bloom’s syndrome. The pathways linking the genetic defect to the eventual development of various neoplasms remain unknown, but the chromosomal instabilities and neoplastic outcomes are related to specific genetic defects in DNA replication, DNA repair, cell-cycle checkpoint, or control of apoptosis. *Ataxia-telangiectasia*, Nijmegen breakage syndrome and *ataxia-telangiectasia*-like disorder are defective in the *ataxia-telangiectasia* mutated (ATM) [56], NBS1 [57,58] and Mre11 [59] genes, respectively. Bloom syndrome and Werner syndrome are defective in the RecQ-like DNA helicases, BLM [60] and WRN [61], respectively. DNA helicases are a highly conserved group of enzymes that unwind DNA.

Fanconi anemia phenotypes that result from hypomorphic mutations in the breast cancer susceptibility gene BRCA2 [62], suggest that defective DNA repair may underlie a fraction of the observed chromosomal instability [63,64]. Although it has been shown that FA does not show radiation hypersensitivity at chromosomal level [65] but exhibits delayed radiation induced DNA damage repair compared to normal cells [57,66].

Sanford *et al*. (1989) [67] investigated the frequency of chromatid aberrations in G2 cells following X-irradiation of cells from normal individuals and those with various genetic disorders such as *ataxia telangectiasia*, Fanconi anemia, Bloom’s syndrome and familial polyposis. They found 2–3 times more radiosensitivity in individuals with genetic conditions predisposing to cancer compared to normal. As a result Sanford *et al*. (1989) [67] proposed a defective DNA repair responsible for higher radiosensitivity and the progression of cancer development. By the use of a G2 assay devised by Sanford *et al*. [68], there are indications that individuals with a strong predisposition to cancer show higher chromosomal aberrations, hence exhibit higher degree of radiosensitivity.

Apart from these rare syndromes, the deficient DNA repair capacity has been proposed to be a predisposing factor in familial and sporadic breast cancer cases [45,69]. Genomic instability has also been described for various hereditary cancers including hereditary breast cancer [49,70]. About 10% of apparently normal individuals and 40% of breast cancer cases show elevated radiosensitivity *i.e*., in the range of AT heterozygotes, linking high radiosensitivity with predisposition to cancer [71]. Radiosensitivity has been extensively studied in breast cancer patients [44,45,71,72,73,74]. In these studies, elevated frequencies of chromatid aberrations, micronuclei and impaired repair of DNA damage following irradiation were observed. Helzlsouer *et al*. (1996) [75] and Patel *et al*. (1997) [76] have shown DNA repair proficiency in breast cancer patients and in their first degree relatives. These studies have shown association between DNA repair proficiency and the risk of cancer. The deficient DNA repair capacity has been proposed to be a predisposing factor in familial and in some sporadic breast cancer cases [44,45,69].

Several reports show an association between prostate cancer risk and genetic variants of genes involved in DNA damage response such as NBS1 [77], ATM [78], and BRCA2 [79]. Peripheral blood leukocytes of prostate cancer patients exposed to various doses of gamma rays did not exhibit radiosensitivity but showed a marked delay in repair of radiation induced DNA damage [46].

More than 50 different types of cancer prone conditions show high levels of radiation induced chromosome damage [80]. In a series of experiments Parshad *et al.* have shown higher G2 radiosensitivity for fibroblasts derived from cancer patients or their relatives compared to normal individuals. [67,80]. These enhanced chromatid aberrations have been attributed to repair deficiency in DNA of these cells [81], that it has genetic basis and also is associated with genetic susceptibility to cancer [68]. High frequency of chromatid type aberrations were detected in radiosenistive cell lines such as AT, SCID and Irs2 defective in DNA damage repair [23,26,82].

To account for these high levels of radiosensitivity, several experiments were performed indicating that the DNA of cancer prone cells repair more slowly than that of normal individuals, or produce more breaks than that of normal cells [45,46,52]. It has also been shown that the type and yield of radiation induced chromosomal aberrations at a given cell cycle stage depends on the combined effect of DNA repair processes and chromatin dynamics [83]. These observations support the findings that cells exhibiting enhanced chromatid radiosensitivity are deficient in DNA repair [67,80]. Moreover, several proteins involved in DNA repair have been shown to produce discrete foci in response to ionizing radiation known as ionizing radiation induced foci (IRIF) or DNA repair foci. Since some residual IRIF remain in cells for a relatively long time after irradiation, there might be a possible link between residual IRIF and cellular radiosensitivity [84].

Variation of inherent radiosensitivity between individuals has also been linked to polymorphisms in single nucleotides [85]. Single nucleotide polymorphisms (SNPs) make up to 90% of the naturally occurring sequence variation in the human genome and SNPs in genes related to the biological response to ionizing radiation [86,87]. A substantial effort has been made during the past 10 years to discover genetic markers, primarily SNPs, associated with variation in the intrinsic response of individuals to radiation and adverse responses to radiotherapy [88]. Genome wide screen based studies identified microsatellite markers associated with acute adverse effects following radiotherapy in cancer patients [89,90]. However, although possible associations between genetic markers and radiosensitivity has been found, strong association between a specific marker or even markers has not yet been established; probably due to inadequate knowledge of the molecular pathology of adverse reactions induced by radiotherapy.

In terms of carcinogenesis, radiosensitivity might potentiate effects of low dose ionizing radiation and increase the frequency of radiation induced cancer. On the other hand it might also potentiate the destroying effects of radiation when used for treatment of tumors, although induced bystander effects cannot be neglected.

## 4. Radioadaptation and Carcinogenesis

Radiation hormesis, also known as adaptive response, is the stimulation, often considered to be beneficial, from low doses of ionizing radiation [91,92]. Deleterious effects of large doses of ionizing radiation are evident but the recognition of this biological response to low doses of ionizing radiation is contradictory to the concept of “all radiation is harmful”. Numerous (thousands) scientific research papers show that low dose irradiation is stimulatory and/or beneficial in a wide variety of microbes, plants, invertebrates, and vertebrates [91,93]. The evidence shows that radiation has a stimulating effect on a number of biological processes and can induce resistance against higher doses of ionizing radiation. During the past three decades, considerable interest has been focused on inducible cellular processes occurring in cells following exposure to low doses of ionizing radiation. Similar to all biological responses of cells to IR induced or exogenous stress [94], this process is also mediated through DNA damage. The changes in cell radioresistance following small dose irradiation are considered to be a small part of all cellular responses to radiation induced stress [95]. The consequences of such a stress could cause accelerated cell division, malignant transformation, hormesis and many other things. Adaptive response is one of the most attractive responses of cells to low dose ionizing radiation.

The adaptive response was first reported by Samson and Carins (1977) [96] in *Escherichia coli* as an inducible form of DNA repair acting on alkylating damage. A similar adaptive response has also been observed in some higher mammalian systems [97] and in lymphocytes exposed to a low dose of ionizing radiation [98,99]. Most of these studies were performed in *in vitro* conditions and there is now little doubt that the *in vitro* pretreatment of human lymphocytes with tritiated thymidine or with low doses of X-rays makes these cells less susceptible to cytogenetic damage by subsequent high acute doses of X- or gamma-rays [4,5,6,99]. This adaptive response to IR, occuring after very low exposures (0.005–0.01 Gy) which by themselves do not induce observable chromosomal aberrations, has been attributed to the induction of a repair mechanism that causes the restitution of X-ray induced chromosome breaks [100].

Adaptive responses can reduce radiation-induced DNA damage, mutagenesis, the frequency of chromosomal aberrations, micronuclei and cell transformants [101]. It was also shown that conditioning of human lymphocytes *in vitro* with 20 mGy of X-rays led to resistance against induction of SSB and DSB, produced by a 1 Gy challenging dose. Persistent DNA strand discontinuities are thought to trigger the signal for adaptation against IR [102]. A similar mechanism is proposed for induction of chromatid type aberrations, following G2 irradiation of cells [103,104].

The induction of a radioprotective mechanism by low doses has important implications for human health. Various *in vivo* studies are also indicative of occurrence of this phenomenon *in vivo*. A significant decrease in sensitivity to bleomycin in the lymphocytes of children internally exposed to 137Cs [105], the induction of the cytogenetic adaptive response by bleomycin and actinomycin D to gamma irradiation in bone marrow cells [106], the induction of the adaptive response in the lymphocytes of hospital workers occupationally exposed to X- and gamma rays [107,108,109,110] and the exhibition of an adaptive response in the human lymphocytes exposed to the fallout from the Chernobyl accident [111], are illustrations of these studies. *In vivo* experiments have shown that low doses of radiation help protect against radiation-induced myeloid leukemia [112] and spontaneous cancer in mice [113].

In a study, Cohen showed that lung cancer deaths decreased with increased radon concentration in homes [114]. Other studies also confirm this benefit from low doses of radon [115,116] which might lead to a reduction in lung cancer. A study showed that the cancer mortality rate of exposed residents of Taipei (Taiwan) in apartments built with cobalt-60 contaminated steel bars, dropped from 50 to 4 per 100,000 people; while that of the general population increased from 82 to 108 per 100,000. The average radiation dose received by residents was about 1.5 cGy/y (the range was 0.1 to 16 cGy/y), about 10 times more radiation than ambient levels in Taipei [117]. Sakamoto also showed that low dose irradiation of the torso was the most effective treatment for malignant lymphoma [118].

These observations and accumulating evidence led the French Academy of Sciences [119] to accept radiation hormesis. Natural occurrence of increased ionizing radiation in certain parts of the world, areas in Brazil, Egypt, Iran, and India have up to 20 times more radiation than the US average of 2 mGy/y [120] might be indicative of the safety of low dose IR. Some believe that a lifetime dose of 5 cGy is not only safe but it has been shown to be healthy by 7 million person years experience with exposure and a carefully selected control of nuclear workers [121], that small doses of ionizing radiation reduce total cancer mortality in both animals and humans [91,121,122]. Using the parameters of cancer mortality rates or mean lifespan in humans, no scientifically acceptable study was found indicating that less than 10 cGy was harmful. Cancer risk after exposure to natural background radiation and medical X-rays is often less than the risk of spontaneous cancer. Therefore, understanding mechanisms underlying the basis for hormetic responses will provide new insights about risks and benefits of low dose ionizing radiation exposure [123].

Although it is thought that ionizing radiation is essential for life and all the criteria of an essential agent are met by low dose irradiation [91,124], there is still fear of low level ionizing radiation [125] and Biological Effects of Ionizing Radiations committees (BEIR) do not support the safety of low dose ionizing radiation [126,127]. Some believe there are political and vested interests behind exclusion of hormesis from the current risk assessment methodology [128].

The reason for these discrepancies might reside in the basic mechanism of induction of adaptation following low dose IR. Studying the adaptive response in the lymphocytes of occupationally exposed individuals showed higher frequencies of chromosomal aberrations (CA) or micronuclei (MN) compared to the control of non-exposed individuals [109,110].

These observations indicate that for the induction of an adaptive response, chromosomal alterations should be produced in cells, even by very low doses of ionizing radiation. These small chromosomal rearrangements might lead to serious health problems, thus, the evaluation of such aberrations might have important implications on risk assessments.

The reduction in MN frequency correlates with a reduction in the neoplastic transformation frequency, suggesting that DSB repair may be enhanced by pre-exposure to low-dose-rate irradiation [129]. The increased capacity for DNA DSB repair (as measured by a reduction in MN frequency) has been shown to be maximally induced in normal human fibroblasts at a dose of 1 mGy of γ-rays, a dose where many cells do not receive one hit, indicating that bystander effects are involved [130,131]. Feinendegen *et al*. [132] indicated that low doses of ionizing radiations can induce the formation of several proteins not found in non-irradiated human lymphocytes [133]. Findings of variability of the radioadaptive response among individuals, suggest genetic determinations [134,135]. Therefore induction of adaptive response may be another form of genome instability due to mutational or epigenetic effects.

It was shown that low doses of IR were capable of protecting human cells deficient in NBS1, ATM, ATR and p53, genes involved in DNA strand breaks repair [136]. Therefore these phenomena may also be related to adaptations in DNA repair processes. Thus, inefficient DNA repair, following suboptimal low dose IR, might lead damaged cells to escape from apoptosis and survive with altered genetic information, eventually expressed as cancer.

However apart from uncertainties surrounding the significance of adaptive responses to DNA damage in tumorigenesis [137], there is now sufficient evidence suggesting the development of cancer models are taking adaptive responses in to account. The possibility of the use of low dose radiation in cancer treatment to improve the outcome of conventional radiotherapy was also raised by various studies which showed increased efficacy in tumor control when low dose irradiation was combined with the ordinary treatment protocol [138]. Adaptive response may also intervene with classical radiotherapy of tumors and lower the efficacy of radiation doses given in each fraction.

## 5. Bystander Effect and Carcinogenesis

The induction of DNA double strand breaks is considered to be the major deleterious consequence of DNA damage, and radiation-induced cell death is attributed to failure or lack of DNA repair, resulting in mitotic failure and/or apoptosis. Unrepaired or misrepaired DNA damage however, might not always be lethal to the cell but show its effects in all descendant cells, *i.e*., the change would be clonally. However, the DNA double-strand break per se does not appear to be involved in initiating genomic instability and a high frequency of radiation-induced instability [139]. Accumulating evidence has shown that multi-cellular responses can be induced in the non-irradiated cells that are in the vicinity of the cells irradiated by low and high Linear Energy Transfer (LET) radiation.

Radiation-induced bystander effects, which have been widely reviewed in the literature [7,140,141,142,143], refer to effects detected in cells that were not directly “hit” by an ionizing radiation track. Implications for carcinogenesis of radiation-induced bystander effects include induction of secondary cancers, induction of genomic instability and delayed or immediate mutations in areas not receiving a direct radiation dose [143,144].

There has been accumulating evidence and an increasing number of studies in over 2 decades, showing that important genetic consequences of radiation may arise in cells that in themselves do not receive direct irradiation. Although the mechanisms underlying bystander effects are fully understood and might involve gap junction mediated cell-cell communication, DNA repair pathways, as well as activation of the p53 damage response pathway. However, this phenomenon has been shown experimentally with various biological end points such as clonogenic assay [145], sister chromatid exchanges [146], micronuclei assay and apoptosis [147,148], as well as altered gene expression and chromosomal instability [140,149,150,151,152,153,154,155,156]. Therefore, the type of bystander response observed, varies depending on the test system being studied and the majority of studies have focused on endpoints that are associated with genomic damage. In all these experimental conditions, DNA double strand breaks are induced in non-targeted neighbors of cells targeted by radiation or in cells grown in culture medium collected from irradiated cells, as shown by the formation of γ-H2AX foci [157,158,159]. However, the number of DSBs induced in directly irradiated and bystander cells was different [160]. Bystander responses have been observed in a range of cell types, following both high and low-LET radiation. The data available suggest that the response predominates at low doses (5 mGy) and saturates at higher doses (5 Gy) [147,161].

Radiation-induced bystander effects are responses, normally associated with directly irradiated cells, observed in un-irradiated cells as a result of receiving signals from irradiated cells [7,140,162,163]. There is evidence that bystander signals can induce genomic instability both *in vivo* [164] and *in vitro* [145,149]. This instability is very frequent and nonclonal, which means that a daughter cell can show a mutation not transmitted by the parent cell. These signaling events might be mediated by the reactions of radiation induced free radicals on DNA, with the existence of a threshold at which the bystander signal is not operative (0.1 Gy dose of X-rays) [151]. On the other hand, the implication of oxidative processes in bystander effects has been shown in many indirect experiments using radical scavengers or antioxidants [154,156,158].

The production of the bystander signal leading to an increase of frequency of micronuclei in non-targeted cells is to some extent independent of the level of DNA damage in the irradiated cells which produce the signal [156]. In some studies bystander signaling molecules and their modulators have concentrated on the influence of DNA damage and the status of DNA repair mechanisms.

It was shown that bystander effect can influence DNA repair pathways (HRR or NHEJ) to alter cellular response from a hyper radiosensitivity state to radioresistance [165]. Radio-adaptive bystander effects were also induced in un-irradiated cells by a transmissible factor (s) present in the medium of cells exposed to different doses of γ-radiation. This radio-adaptive bystander effect was correlated with a reduced cellular level of protein p53 and an increase of intracellular reactive oxygen species and enzymes involved in DNA repair [166].

Activation of repair mechanisms has also been shown to be cell cycle- and radiation dose-dependent [167]. In their study Rothkamm and Lobrich noticed that in non-dividing primary human fibroblasts irradiated by very low doses (~1 mGy) of IR, unrepaired double strand breaks persist for many days, whereas irradiation with higher doses, induced efficient and fast double strand break repair [167]. These phenomena observed after low dose irradiation are closely related to bystander effects, which can be produced with low doses of X-irradiation from 1.2 to 5 mGy in primary human fibroblasts [168].

As malignant transformation is generally regarded as being initiated by a gene mutation or a chromosomal aberration, the initiating lesion for malignant transformation has been similarly attributed to DNA damage in the directly irradiated cell. Thus, the evaluation of radiation health risks and radiation protection practice has been based on such direct (targeted) effects of radiation. The predominant interests about the radiation-induced bystander effect, focus on the cancer risk assessment of low-dose radiation [169].

Carcinogenesis and radiation risk management is such that at low doses, the response to radiation becomes important, in terms of how much bystander factor is produced, how far it travels and what effect it has. The bystander effect mostly increases the size of the cellular population “at risk” of radiation induced damages. One consequence of radiotherapy is an increased risk of initiation of new carcinogenic processes and development of further primary cancers; the cumulative incidence of second primary malignancies in patients treated by radiotherapy is estimated to be as high as 20% [170,171,172]. The conditions under which radiation induced bystander effects are induced *in vivo* will ultimately determine their impact on radiation induced carcinogenic risk [173,174]. On the other hand, the pro-mitogenic effects observed in some systems [151,175,176] probably influence the results of radiotherapy through an increase in survival of targeted cells due to bystander adaptation effects leading to increased risk of tumor re-growth and modulation of potential targets in radiotherapy [172].

Genomic instability and the probable consequence of bystander effects, indicates nonlinearity into the low-dose area. The observation that bystander cells exhibit genome instability similar to directly hit cells with propagation of heritable DNA alterations to cells occurring many generations after radiation exposure, indicates that cellular response to low dose ionizing radiation is a complex interplay of various modulating factors [177]. If carcinogenesis does not result from directly induced DNA mutations, then the carcinogenic initiation process may not simply relate to radiation dose [143]. Therefore the response of the tissue/cell population rather than the dose of radiation will determine the fate of the cell and the tissue.

## 6. Distant Bystander (Abscopal) Effect

It is sometimes observed in cancer therapy that there is a beneficial side effect of radiation therapy, the so-called “abscopal effect”. The term “abscopal effect” caused by radiotherapy was first introduced by Mole in 1953 [178] and was derived from the Latin “ab” (*i.e*., “position away from”) and “scopos” (*i.e*., “a target for shooting at”). This term was used to describe systemic effects that were observed at non-irradiated sites in an animal, after treatment with localized radiotherapy.

There is now accumulating case reports describing the abscopal effect occurring in various malignancies following radiotherapy. These malignancies include lymphoma [179,180,181], papillary adenocarcinoma [182], melanoma [183], adenocarcinoma of the esophagus [184], chronic lymphocytic leukemia [185], hepatocellular carcinoma [186], toruliform para-aorticlymph node metastases of advanced uterine cervical carcinoma [187] and lung metastases of hepatocellular carcinoma [188]. The definition of the abscopal effect has been broadened to include other forms of local therapy that have systemic effects, *i.e*., a distant bystander effect [189,190,191]. It has been shown that fractionated, but not single-dose radiotherapy, induces an immune-mediated abscopal effect when combined with the anti-CTLA-4 antibody [192].

The abscopal effect apparently operates through immune mediated [193] or p53 mediated mechanisms [194]. Recent report by Kinashi *et al*. (2010) [195] regarding reduction of micronuclei in distant splenic T-lymphocytes, following head irradiation by ascorbic acid, might be indicative of involvment of DNA repair machinery in the abscopal effect. The abscopal effect as a non-targeted radiation effect might be correlated with epigenetic mechanisms especially through DNA methylation and small RNAs induced in directly exposed or bystander tissues [196]. However, the abscopal effect is a controversial issue [197] and there is a need for more experimental approaches for verification of the effect itself and possible relevant mechanisms.

## 7. Conclusions

**Figure 1:**
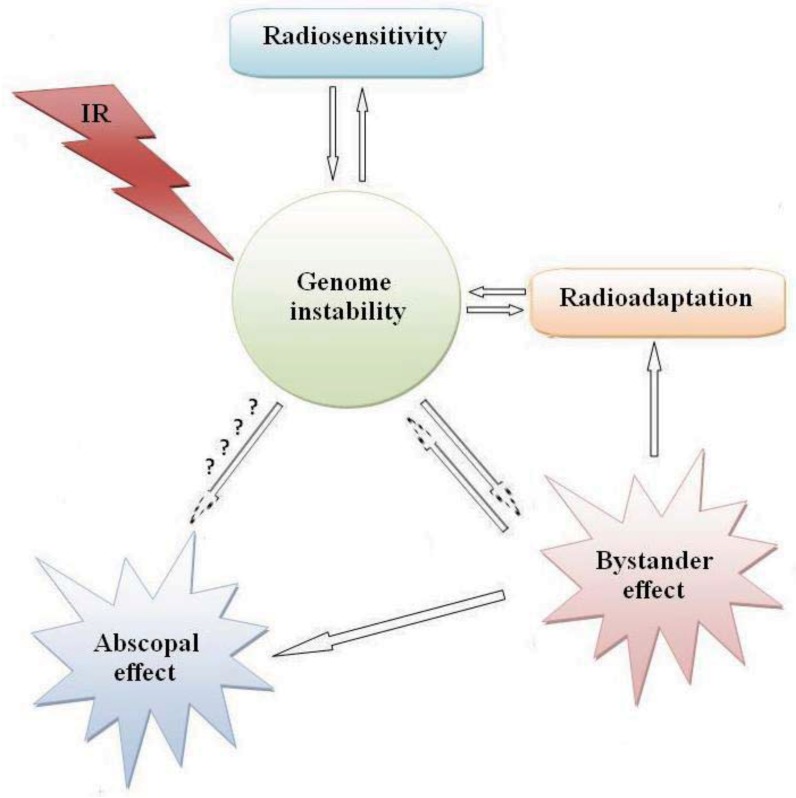
Possible inter-relationships between cellular responses to ionizing radiation.

It appears that the observed low-dose radiation responses (bystander effect, hormesis, and adaptive responses) which follow a non-linear radiation response may depend on the type of radiation, the cell and tissue type and the genetic status of the cells. Another low-dose non-linear radiation response, the phenomenon of low-dose radiation hypersensitivity, can also be added to these categories [198,199] which have been observed in many cell types. It is difficult, therefore to correlate radiation induced carcinogenesis to the cells directly hit by radiation and the induction of DNA double strand breaks. Radiosensitivity, adaptive response, and bystander effects are key players in radiation induced carcinogenesis. All these phenomena apparently involve the same genetic background and signaling pathways; mainly genomic instability (Figure 1).

Although the primary aim in radiotherapy is to increase DNA damage in tumor cells, the ROS produced in radiotherapy threaten the survival of normal cells and induce damage in non-target cells, leading to regrowth or induction of secondary tumors. However, because of the existence of cellular hypo- and hyper-genomic stability states [199,200] the impact of low dose ionizing radiation in carcinogenesis has yet to be determined.
